# Extracorporeal blood purification benefits in post-caesarean patient with severe acute respiratory distress syndrome due to miliary tuberculosis: a case report

**DOI:** 10.1186/s13256-023-03853-w

**Published:** 2023-04-12

**Authors:** Gumarbio Setiadi Zakaria, Muhammad Azhari Taufik, Sidharta Kusuma Manggala, Jennefer

**Affiliations:** 1 Department of Anesthesiology and Intensive Care, Faculty of Medicine, Universitas Indonesia, Cipto Mangunkusumo National General Hospital, Jakarta, Indonesia; 2 Department of Anesthesiology and Intensive Care, Fatmawati General Hospital, Jakarta, Indonesia

**Keywords:** Miliary tuberculosis, Blood purification, Continuous veno-venous hemofiltration

## Abstract

**Background:**

Miliary tuberculosis is a life-threatening disease caused by the hematogenous spread of *Mycobacterium tuberculosis*. It is uncommon in pregnancy. Mortality rates for patients with miliary tuberculosis who require mechanical ventilation are high (60–70%).

**Case presentation:**

We reported a rare and challenging case, a 35-year-old Asian woman with 34 weeks of pregnancy, and miliary tuberculosis with acute respiratory distress syndrome and septic shock. The patient presented with severe acute respiratory distress syndrome, necessitating mechanical ventilation, vasopressor, and pregnancy termination with caesarean section. The patient underwent blood purification with continuous veno-venous hemofiltration using an oXiris filter for 24 hours. After continuous veno-venous hemofiltration, the patient’s condition was greatly improved, and the patient was successfully extubated and was able to breathe spontaneously without vasopressor on the third day. High levels of interleukin-6, interleukin-10, procalcitonin, C-reactive protein, interferon-γ, and tumor necrosis factor-α were found postoperatively.

**Conclusion:**

The bacterial infection of tuberculosis, acute respiratory distress syndrome, and the stress response from the caesarean section contributed to the high levels of cytokines, which correlated with the patient’s severe inflammatory condition. The cytokine levels were greatly reduced after the blood purification procedure and this might be associated with the patient’s clinical improvement. Extracorporeal blood purification could help to disrupt the vicious cycle of inflammation.

## Background

According to World Health Organization (WHO) in 2020, 10 million people worldwide were infected with tuberculosis (TB), including 5.6 million men, 3.3 million women, and 1.1 million children; this number was expected to increase [1]. Indonesia was the second greatest contributor to new cases in 2019–2021. The incidence of TB cases in Indonesia is more than 500,000 new cases per year [2].

Miliary TB is a life-threatening infection caused by *Mycobacterium tuberculosis* with hematogenic spread [3]. From earlier meta-analysis of 13 studies, there were higher odds [odds ratio 4.1, 95% confidence interval (CI) 0.65–25.2] of mortality in pregnant women with active TB compared with those without TB [4]. The incidence of miliary TB in pregnancy is usually related to intravenous drug abuse, malignancy, alcoholism, or human immunodeficiency virus (HIV) [5]. Miliary TB could induce acute respiratory distress syndrome (ARDS) and systemic inflammatory response syndrome (SIRS). TB is reported as the etiology of 3.6% of ARDS cases [6].

The diagnostic delay might adversely affect for the patients’ clinical prognosis. The severe infectious condition could lead to sepsis [7]. According to the WHO, sepsis remained as the most common cause of death in 2020, accounting for one in every five fatalities, with 11 million deaths per year [1].

We presented an unusual case of a pregnant woman with miliary TB infection that progressed to septic shock from pneumonia. We highlight the advantages and beneficial outcomes of blood purification in this case.

## Case presentation

A 35-year-old Asian woman was admitted to the hospital with a history of shortness of breath and cough, accompanied by phlegm production, for more than 3 weeks. The patient was 34 weeks pregnant. At the initial physical examination, the patient looked seriously ill with blood pressure of 140/90 mmHg, heart rate of 130 beats per minute, respiratory rate of 28 breaths per minute spontaneously, body temperature of 37.9 °C, and oxygen saturation of 98% with 8 L per minute oxygen administered via a simple face mask. The patient had a history of cardiac arrhythmias (paroxysmal supraventricular tachycardia type) and underwent ablation in 2019, without rate control medication afterward. The patient also had a previous history of caesarean section surgery in 2016 and a history of two spontaneous abortions. The patient had unknown history of TB contact and had completed Bacillus Calmette–Guérin (BCG) vaccine.

The initial laboratory results showed low hemoglobin, leukocytosis with increased neutrophil–lymphocyte ratio (NLR), elevated liver enzymes, slightly low albumin, hyponatremia, elevated d-dimer levels, and elevated inflammation and infection markers. Severe acute respiratory syndrome coronavirus 2 (SARS-Cov-2) polymerase chain reaction (PCR) swab showed a negative result. Hepatitis B surface antigen (HBsAg), anti-hepatitis C virus (HCV), and anti-HIV examination also showed negative results. From the blood gas analysis, the PaO_2_/FiO_2_ (P/F) ratio was 141. Culture tests were also done upon the patient’s arrival. Chest x-ray examination presented a consolidation and miliary infiltrates in both lung fields. GeneXpert molecular examination showed positive results without any resistance to rifampicin. Abdominal ultrasonography revealed a pregnancy and normal intraabdominal organs. The fetal heart rate was observed at 150 beats per minute. Echocardiography results also presented good results, with good left ventricle (LV) and right ventricle (RV) contractility, with an ejection fraction (EF) of 59%.

The patient was 34 weeks pregnant and diagnosed with sepsis and ARDS from miliary TB and pneumonia. The patient was transferred to the intensive care unit (ICU). The management of the patient included the administration of antibiotics (meropenem 2 g every 8 h), antituberculosis medications [rifampicin 600 mg once daily, ethambutol 1000 mg once daily, isoniazid (INH) 400 mg once daily], oxygen with a high-flow nasal cannula, steroid (dexamethasone 6 mg twice daily for 2 days), and other supportive care.

During the treatment, the patient’s condition worsened, with an increase in respiratory rate of 32 breaths per minute, heart rate up to 140 beats per minute, and body temperature of 38.2 °C, along with a decrease in P/F ratio to 98.4, and fetal distress with heart rate over 160 beats per minute. The pregnancy was terminated through caesarean section surgery under general anesthesia and intubation. Intraoperatively, the blood pressure began to drop, necessitating vasopressor norepinephrine at a dosage of 0.2 µg/kg/minute.

The patient was mechanically ventilated under sedation in ICU after the surgery. The P/F ratio was 90, with FiO_2_ of 60%, positive end-expiratory pressure (PEEP) of 8 cm H_2_O, and high P-plateau of up to 36 cm H_2_O, despite the target of tidal volume was already 5–6 cc/kg. The chest x-ray examination showed an increase of infiltrate in both lung fields. The laboratory results showed increasing levels of leukocytes, NLR, C-reactive protein (CRP), and procalcitonin (PCT), along with cytokine levels. Her sequential organ failure assessment (SOFA) score was 8, and acute physiology and chronic health evaluation (APACHE) II score was 13.

We decided to perform continuous veno-venous hemofiltration (CVVH) with an oXiris filter for nonrenal indication. The patient showed severe SIRS clinical condition, unstable hemodynamic with gradual vasopressor increase, and substantial elevation of cytokine levels. The patient’s urine output was still around 1–2 cc/kg/hour with no elevation of urea and creatinine level. CVVH was performed for 24 hours with a blood flow rate of 150–180 cc/minute, post-filter replacement fluid of 1500 cc/hour, removal of 0, and continuous heparin administration of 150 units/hour.

After 3 hours of ongoing CVVH, the P-plateau gradually decreased from 36 to 29. The hemodynamic began to improve, marked by a decrease in the heart rate to around 105 beats per minute, as well as a reduction of norepinephrine dose. On the second day of postoperative and post-CVVH, the patient’s clinical condition was well improved. The hemodynamic was stable without vasopressor, minimal support of ventilator, with PEEP of 6, and the P/F ratio had improved to 247.5. The laboratory results showed improvements (Table 1), along with the decreased levels of cytokines (Table 2), and also with improvement of inflammation and infection markers. Electrolytes, SGOT, SGPT, and bilirubin were within normal limits. The blood, sputum, and urine cultures showed no signs of bacteria or yeast.

**Table 1:** The laboratory results

Parameters	Value			Normal value
	Preoperative	Postoperative (before CVVH)	After CVVH	
Hemoglobin (g/dL)	9.5	9.8	10.2	11.7–15.5
Leukocytes (× 10^9^/L)	13.7	14.9	11.5	5.0–10.0
Platelets (× 10^9^/L)	385	487	313	150–440
Absolute lymphocytes (× 10^9^/L)	1.29	0.94	1.38	0.7–4.6
NLR	5.6	13.7	6.2	4.0
SGOT (U/L)	128	133	31	8–45
SGPT (U/L)	126	139	43	7–56
Urea (mg/dL)	11.7	24.9	48.6	16.6–48.5
Creatinine (mg/dL)	0.36	0.27	0.32	0.51–0.95
Albumin (g/dL)	3.29	–	3.82	3.5–5.2
PT (seconds)	13.4	14.6	13.1	11.7–15.1
APTT (seconds)	29.6	32.9	30.8	24.8–34.4
d-dimer (ng/mL)	5982	3559	2095	≦ 500
Sodium (mmol/L)	131	130	142	136–145
Potassium (mmol/L)	3.7	4.0	3.5	3.5–5.1
Chloride (mmol/L)	96	97	100	98–107
Magnesium (mmol/L)	–	1.5	–	1.6–2.6
Calcium ion (mmol/L)	–	1.12	–	1.00–1.15
Blood glucose level (mg/dL)	152	145	101	70–140
CRP (mg/dL)	13.08	28.4	6.55	≦ 0.5
PCT (ng/mL)	0.97	1.86	0.07	< 0.5
Lactate (mmol/L)	2.6	3.0	1.7	0.5–2.2

*NLR* neutrophil to lymphocyte ratio, *SGOT* serum glutamic oxaloacetic transaminase, *SGPT* serum glutamic pyruvic transaminase, *PT* prothrombin time, *APTT* activated partial thromboplastin time, *CRP* C-reactive protein, *PCT* procalcitonin

**Table 2:** Cytokine levels before and after continuous veno-venous hemofiltration

Parameters	Value (pg/mL)		Normal value
	Before CVVH	After CVVH	
IL-6	14.42	7.84	0.7–12.5
IL-10	12.66	3.51	< 5.0
TNF-α	76.16	20.04	< 8.8
IFN-γ	355.29	15.1	< 5.0

*IL* interleukin, *TNF* tumor necrosis factor, *IFN* interferon

On the 3rd day, the patient was successfully extubated. The SOFA score and APACHE II score improved to 2 and 4, respectively. Pyrazinamide 1000 mg once daily was added to the antituberculosis medications. The patient’s clinical condition improved gradually. The patient was transferred to the ward 3 days later and discharged from the hospital several days later, in good condition.

## Discussion

The patient was diagnosed with ARDS caused by miliary TB on the basis of her clinical condition and supporting examination. It can be challenging to diagnose TB in pregnancy. Besides the unusual symptoms, the delay of radiological examination (for example chest x-ray) can lead to delayed diagnosis [5, 8]. This can further lead to therapy delays, thereby increasing the risk of TB infection becoming hematogenic and developing into miliary TB. Several examinations are used to diagnose TB, including radiology such as chest x-rays and computed tomography (CT)-scans, culture analysis to identify TB pathogens, tissue biopsies via bronchoscopy, and PCR examination from bronchoalveolar lavage samples. Each of these modalities has a different positivity rate [7].

Miliary TB is a specific type of disseminated TB resulting from lymphatic and hematogenic spread from the initial TB infection site and it can be potentially fatal if left untreated. However, mortality increases when ARDS, septic shock, and multiple organ failure occur. ARDS most commonly occurs as the result of miliary disseminated TB [9]. Lee *et al.* reported that 46% of the nonsurvivor group of patients with TB and ARDS experienced septic shock [10].

The severe SIRS condition in the patient was due to miliary TB, sepsis, and pregnancy. As we know, this is closely related to pathogen-associated molecular patterns (PAMPs) and damage-associated molecular patterns (DAMPs). Lipoarabinomannan, a component of mycobacterial cell wall, similarly stimulates the inflammatory cascade as antigens in bacterial sepsis do, such as lipopolysaccharides [11]. The stress response due to caesarean section in this patient also resulted in tissue damage, aggravating the SIRS condition caused by DAMPs.

From our perspective, the crucial interventions for this patient were pregnancy termination by caesarean section, the administration of antibiotics and antituberculosis medications, vasopressors, the management of mechanical ventilation for ARDS, and lastly, CVVH.

Broad-spectrum antibiotics and antituberculosis medications should be administered as soon as feasible once the diagnosis is established. Early diagnosis and management tend to provide better outcomes. Singh *et al.* stated that delaying the administration of antituberculosis medications might have an adverse influence on the patient’s prognosis [6].

**Fig. 1:**
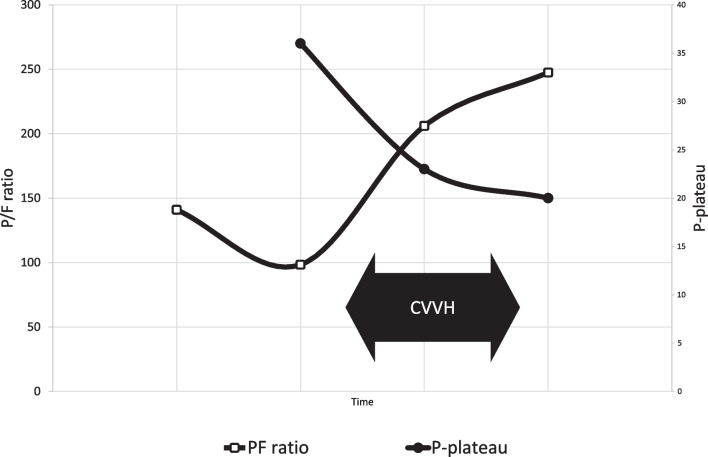
P/F ratio and P-plateau improvements

The CVVH was being conducted as blood purification (for nonrenal indication) in this patient. Severe SIRS and ARDS conditions in this patient were concomitant with elevated cytokine levels. The patient’s hemodynamic was markedly improved, as shown by the improvement of mean arterial pressure (MAP) and the reduction of vasopressor dose requirement. Figure 1 showed the improvement of ARDS condition as the P/F ratio improved and the P-plateau decreased. The organ function improvement was shown due to lower SOFA and APACHE II scores.

**Fig. 2:**
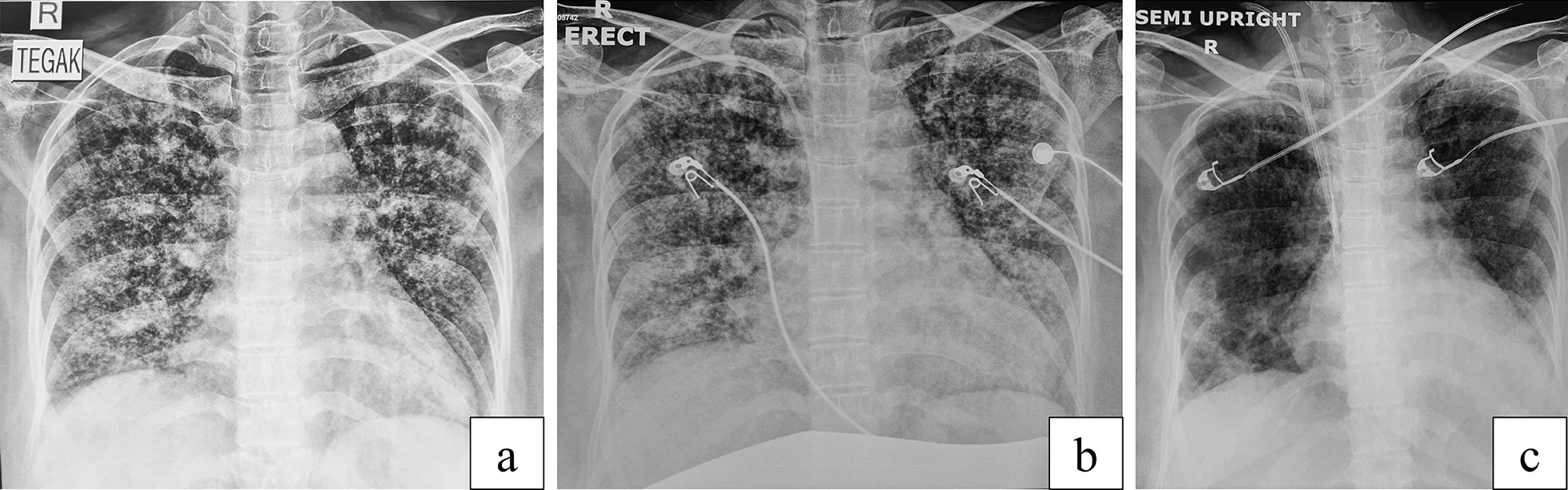
Chest x-ray when admitted (**a**), before continuous veno-venous hemofiltration (**b**), and after continuous veno-venous hemofiltration (**c**)

Figure 2 also showed the significant improvement in the patient’s chest x-ray result. The patient’s consolidation and miliary infiltrate in both lung fields were much diminished after CVVH. The clinical improvements and radiological findings were also consistent with decreased levels of cytokines, such as IL-6, IL-10, TNF-α, and IFN-γ, along with improvement of inflammation and infection markers. Several journals also reported how blood purification has been proven to reduce cytokine levels in sepsis and SIRS [12–14].

We performed CVVH using oXiris filter, which is a heparin-coated hemodiafilter, highly biocompatible, and widely known for its effectiveness in supporting kidney function. However, the device may also be used to eliminate cytokines and endotoxins in a nonselective approach. The filter has three layers of membrane: the heparin graft membrane, the polyethyleneimine (PEI) surface layer in the center, and the AN69 membrane at the base. The combination provides anticoagulation, renal supports, and endotoxins and cytokines removal [15, 16].

Despite no renal indication, the CVVH procedure was done to reduce cytokine levels, so as to improve the SIRS and ARDS conditions in the patient. The CVVH can also assist in preventing worsening of the condition and improving the outcome.

The substantial reduction in cytokine levels could prevent the development of acute kidney injury (AKI). According to several studies, elevated levels of cytokine and endotoxins in sepsis condition could induce AKI and possibly fatality [17, 18].

The improvement of the patient’s clinical condition was clearly visible before and after CVVH, especially the improvement of ARDS condition radiologically, and also visible from P/F ratio and P-plateau parameters. The hemodynamic improvement was marked by the decrease of norepinephrine dose. Lastly, the levels of cytokines, and inflammation and infection markers were also improved after CVVH. Other organ damage can be minimized and prevented by reducing cytokine levels and endotoxins [19, 20].

The role of CVVH as blood purification in sepsis is still a subject that is being researched and addressed today. Particularly in terms of whether individuals with sepsis might benefit from blood purification. The role of CVVH in sepsis is closely related to the widely recognized pathophysiology of the infection and inflammatory processes [21].

The initial stage of infection is immune system activation brought on by pathogen recognition. The surface pattern recognition receptor (PRR) on the cell surface of immune cells detects PAMPs, such as lipopolysaccharide, an endotoxin produced by Gram-negative bacteria, lipoteichoic acid, and bacterial deoxyribonucleic acid (DNA) or ribonucleic acid (RNA) fragments, during infection [21]. The activation of leukocytes by this signal results in the production of pro- and antiinflammatory cytokines, including tumor necrosis factor-alpha, IL-1, IL-6, IL-8, and IL-10. A cytokine storm, or large-scale release of cytokines into the blood, is regarded to be the root cause of major organ malfunction [22].

In addition to the PAMPs mechanism, the DAMPs mechanism is also presented and discharged by the injured host cells. Mitochondrial DNA, uric acid, amyloid-β, S100 proteins, extracellular adenosine triphosphate (ATP), high-mobility group box (HMGB) 1 protein, heat-shock proteins (HSPs), and extracellular matrix proteins are a few examples of recognized DAMPs [23]. When PRR detects these DAMPs, the immune system is then activated, which sets off a cycle of inflammation that eventually results in sepsis and a cytokine storm [21].

There are various theories regarding how CVVH might contribute to sepsis or septic shock. The first idea holds that by eliminating PAMPs and DAMPs that encourage leukocyte activation and cytokine release, the systemic inflammatory cascade could thereafter be inhibited. Second, they might also lower cytokine concentrations below a dangerous threshold to restrict the harmful effects of cytokines locally. The third explanation is to promote leukocyte chemotaxis toward infected tissue, which has a higher concentration of cytokines than other locations [16].

The advantages of this blood purification have been documented in numerous research investigations. One is from retrospective cohort research by Schwindenhammer *et al.*, which found that for the most severe patients, survival was better than predicted by a severity score [simplified acute physiology score (SAPS II)] while using the oXiris hemofilter. Moreover, particularly in patients with intraabdominal infections or Gram-negative bacterial infections, the hemodynamic state and lactatemia appeared to recover quickly [24]. In a randomized controlled trial, Hawchar *et al.* found that extracorporeal blood purification using Cytosorb for 24 hours in the early stages of septic shock is safe and has some advantages, as evidenced by decreases in norepinephrine requirements, serum PCT, and big endothelin (BigET)-1 levels [25].

Putzu *et al.* performed a meta-analysis of 11 randomized trials on blood purification with CVVH in patients with severe sepsis/septic shock or ARDS, without renal failure, needing renal replacement therapy and discovered that patients who received blood purification with CVVH had significantly lower mortality compared with conventional therapy without blood purification [OR = 0.58 (95% CI 0.42, 0.81)], *P* = 0.002). Also, it appeared that the CVVH group’s mechanical ventilation and ICU hospitalizations were shorter [26]. However, a meta-analysis and trial sequence analysis (TSA) on a total of 39 randomized controlled trials that used blood purification therapy in patients with sepsis, conducted by Snow *et al.*, discovered that pooled data from conventional meta-analysis of prospective randomized controlled trials showed that CVVH, endotoxin adsorption devices, and nonspecific adsorption devices are linked with higher survival among patients with sepsis in the ICU. But in the end, the TSA found insufficient evidence to support this since the sample size was too small [27]. Based on these, we will undoubtedly require more high-quality randomized controlled studies in the future, with significant sample sizes and adequately powered for mortality, to ascertain the true impact of CVVH on sepsis.

## Conclusion

The patient’s clinical improvement could not be achieved without appropriate management in the ICU. By lowering the cytokine levels, and thus interrupting the cascade of inflammation, blood purification with CVVH is thought to assist patients with severe SIRS, sepsis, or ARDS. Due to recent studies’ contradictory findings about the significance of CVVH in severe SIRS or sepsis, more research is undoubtedly required.

## Data Availability

The data that support the findings of this study are not publicly available. Data are, however, available from the authors upon reasonable request and with permission.

## Abbreviations

AKI: Acute kidney injury
APACHE: Acute physiology and chronic health evaluation
APTT: Activated partial thromboplastin time
ARDS: Acute respiratory distress syndrome
ATP: Adenosine triphosphate
COVID-19: Coronavirus disease 2019
CRP: C-reactive protein
CVVH: Continuous veno-venous hemofiltration
DAMP: Damage-associated molecular patterns
DNA: Deoxyribonucleic acid
HCV: Hepatitis C virus
HIV: Human immunodeficiency virus
HMGB: High-mobility group box
HSP: Heat-shock protein
ICU: Intensive care unit
IFN: Interferon
IL: Interleukin
INH: Isoniazid
MAP: Mean arterial pressure
NLR: Neutrophil to lymphocyte ratio
PAMP: Pathogen-associated molecular patterns
PCR: Polymerase chain reaction
PCT: Procalcitonin
PEEP: Positive end-expiratory pressure
PEI: Polyethyleneimine
PT: Prothrombin time
RNA: Ribonucleic acid
SARS: Severe acute respiratory syndrome
SGOT: Serum glutamic oxaloacetic transaminase
SGPT: Serum glutamic pyruvic transaminase
SIRS: Systemic inflammatory reaction syndrome
SOFA: Sequential organ failure assessment
TB: Tuberculosis
TNF: Tumor necrosis factor
TSA: Trial sequence analysis
WHO: World Health Organization
